# Skeletally immature patient showed lower graft maturity than skeletally mature patient after ACL reconstruction with a rounded rectangular femoral tunnel

**DOI:** 10.1038/s41598-021-99532-1

**Published:** 2021-10-07

**Authors:** Kazuki Asai, Junsuke Nakase, Kengo Shimozaki, Rikuto Yoshimizu, Mitsuhiro Kimura, Hiroyuki Tsuchiya

**Affiliations:** Department of Orthopaedic Surgery, Graduate School of Medical Science, Kanazawa University, 13-1 Takara-machi, Kanazawa-City, 920-8641 Japan

**Keywords:** Medical research, Outcomes research

## Abstract

To compare the clinical results and ligamentization of anterior cruciate ligament reconstruction (ACLR) between skeletally immature and mature patients. Two-hundred-and-two patients who underwent primary ACLR were evaluated retrospectively. The clinical outcomes were compared between skeletally immature (immature group 1, n = 27) and mature (control group 1, n = 175) groups. Graft ligamentization of the reconstructed anterior cruciate ligament (ACL) using magnetic resonance imaging (MRI) signal intensity at 6 months postoperatively was compared between immature group 2 (n = 16), which included participants from immature group 1, and control group 2 (n = 32), created by recruiting data-matched controls from control group 1. Immature group 1 had significantly higher revision (14.8%) and pivot shift test positive (22.2%) rates than control group 1 (2.9% and 4.0%, respectively) (*P* = 0.020 and 0.003, respectively). The signal intensity in immature group 2 were significantly higher at the mid-substance and distal site of the reconstructed ACL than those in control group 2 (*P* = 0.003 and 0.034, respectively). Skeletally immature patients had higher graft revision and residual rotational laxity rates. Reconstructed ACL in skeletally immature patients showed higher signal intensity on MRI at 6 months postoperatively.

## Introduction

The incidence of pediatric anterior cruciate ligament (ACL) injuries is increasing. This may be related to increased participation in sports, early sport specialization, or increased recognition of ACL injuries^1,2^. Between 1994 and 2006, there was a 924% increase in the performance of ACL reconstruction (ACLR) among patients less than 15 years of age in the United States^1^. Shaw et al. reported a 147.8% increase in the incidence of ACL injuries in patients less than 15 years of age from 2005 to 2015^2^. ACLR is performed in pediatric patients to restore sufficient knee stability to allow them to return to sports as well as to prevent secondary lesions such as meniscus and cartilage injuries, which can lead to premature osteoarthritis^3^. However, graft failure after ACLR is a known complication, especially in immature patients. Geffroy et al. reported re-tears in 9% of patients with open growth plates and 2.8% of those with closed growth plates^4^. There are several studies concerning the surgical techniques, type of grafts, postoperative rehabilitation and time to return to sport with ACLR in skeletally immature or adolescent patients; however, very few studies have compared ligamentization of the reconstructed ACL between the mature and immature groups^5–7^. Further, poor outcomes following ACLR in skeletally immature knees, including higher revision rates, continue to be a problem.

Three stages of graft maturity as part of ligamentization are generally described: initial avascular necrosis, second revascularization, cell repopulation and resynovialization, and finally, remodeling^8^. Scheffler et al. reported that mechanical properties of transplanted grafts decrease during the postoperative ligamentization process and never return to normal^9^. Graft signal on magnetic resonance imaging (MRI) is a useful tool to qualitatively assess graft ligamentization postoperatively^10^. The volume of revascularization tissue influences the MRI signal intensity of the graft, particularly within the first 2 postoperative years^11^. A study has reported an initial increase in the graft signal intensity starting from the third postoperative month, with peak signals at 4–6 months, followed by a decrease^12^. In an animal study, a higher graft signal intensity during the initial phase was followed by a signal decrease that correlated with improvement in the mechanical properties of the graft^13^. Only a few studies have evaluated ligamentization in skeletally immature patients following ACLR^14^. Pauvert et al. demonstrated that ligamentization of the reconstructed ACL in pediatric patients with open growth plates is a very slow process with changes continuing even after 2 years postoperatively, as seen on MRI; maturation of the graft at 2 years may be doubtful since the MRI signals at that point do not resemble those of a normal ACL^14^. However, to the best of our knowledge, no studies have used MRI to compare the ligamentization of reconstructed ACL between skeletally immature and mature patients.

In this study, the patients underwent ACLR with a rounded rectangular femoral tunnel using an original rounded rectangular dilator, which was designed to enable a more anatomical and wider tendon-bone junction^15,16^. Zhao et al. demonstrated that an oval-shaped bone tunnel accelerates tendon-bone healing in the early period after ACLR more than a conventional round tunnel in a rabbit model^17^. Wen et al. reported the clinical outcomes of the oval-shaped femoral tunnel technique, which resulted in significantly better knee function and knee laxity restoration and more mature ACL grafts than the conventional round femoral tunnel technique^18^. However, to the best of our knowledge, no study has compared the outcomes of the rounded rectangular femoral tunnel technique between skeletally immature and mature patients.

Therefore, the main purpose of this study was to compare the ligamentization of the reconstructed ACL with a rounded rectangular femoral tunnel using MRI signal intensity between skeletally immature and mature patients at 6 months following surgery. Further, we also attempted to assess the outcomes following ACLR with a rounded rectangular femoral tunnel in skeletally immature patients. We hypothesized that the signal intensity of the reconstructed ACL in skeletally immature patients may be higher than that in skeletally mature patients, implying that ligamentization in skeletally immature patients is slower.

## Methods

### Participants

This study was approved by the Medical Ethics Committee of the Kanazawa University Advanced Science Research Center (1842-1), and informed consent was obtained from each patient or their parent if patients aged < 18 years. All methods were performed in accordance with the relevant guidelines and regulations. A total of 236 patients who underwent primary ACLR by using hamstring tendon (HT) grafts in a single institution between July 2013 and August 2019 were evaluated retrospectively. The mean and minimum follow-up times were 23 ± 9 months and 12 months, respectively. Exclusion criteria were ACLR with bone-patellar tendon-bone grafts, multi-ligament injuries requiring other ligament surgery, and a history of surgery or trauma around the knee joint. A total of 202 patients were included for the analysis after excluding 34 patients. Patients were divided into two groups based on the epiphyseal plate of the distal femur, as confirmed on MRI.

**Figure 1:**
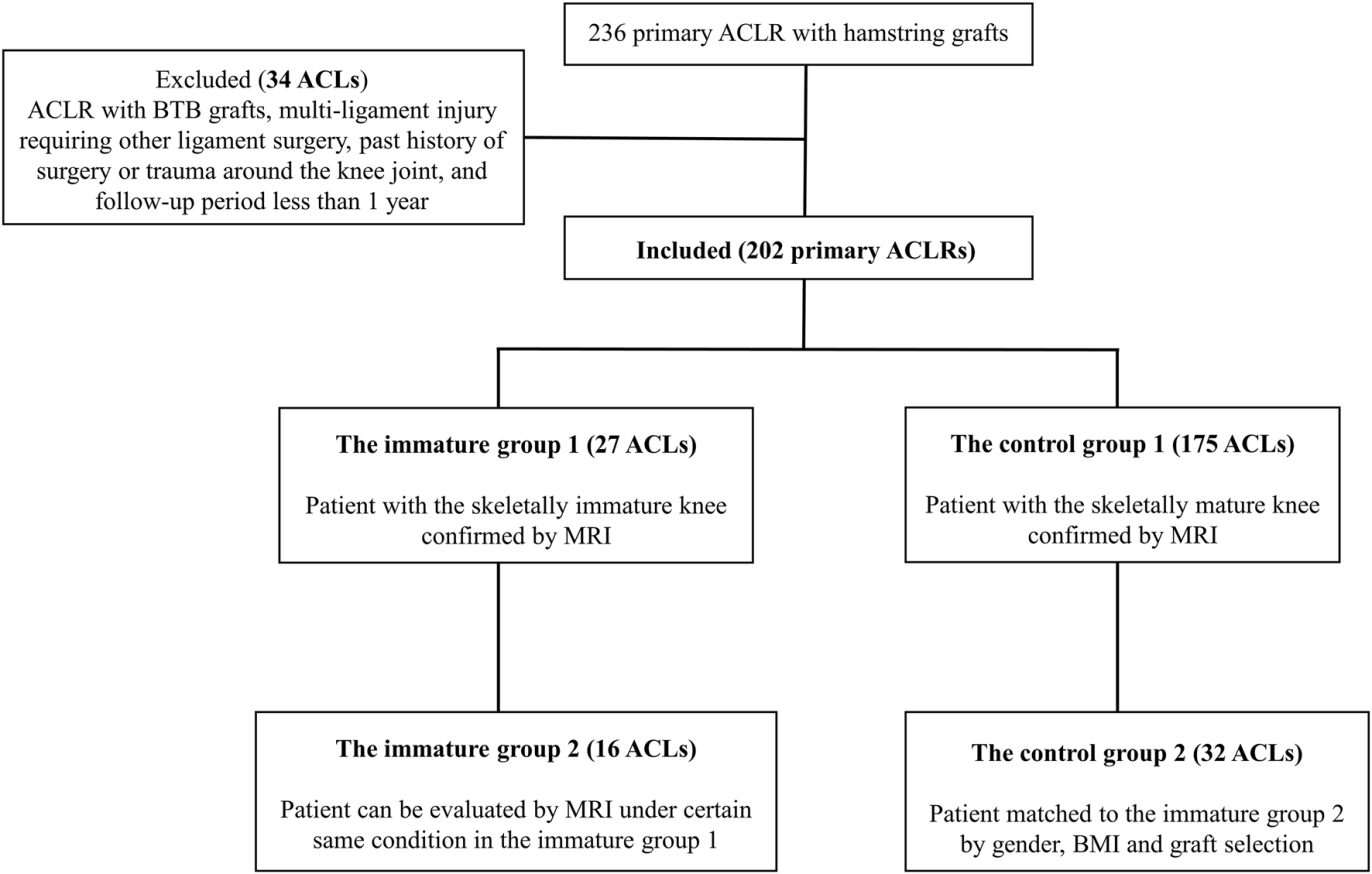
Study flowchart. *ACLR* anterior cruciate ligament, *BTB* bone-patellar tendon-bone, *MRI* magnetic resonance imaging, *BMI* body mass index.

Based on a previous study, knees were categorized as skeletally immature if the epiphyseal plate was > 1.5 mm in thickness or on identifying a multilaminar appearance in the femur on MRI (T2-weighted fat-restraint coronal image)^19^. Accordingly, there were 27 patients in immature group 1 (age, 13.9 ± 1.2; male, 11 and female, 16) and 175 patients with skeletally mature knees in control group 1 (age, 26.2 ± 12.1; male, 85 and female, 90) (Fig. 1). Based on Dedouit’s classification, there were seven patients in stage 1 and 20 patients in stage 2 in immature group 1^19^. We compared the postoperative outcomes following ACLR between immature group 1 and control group 1.

To compare graft ligamentization of the reconstructed ACL based on MRI signal intensity at 6 months postoperatively, we created an immature group 2 (age, 13.6 ± 1.1; male, 8 and female, 8) by including 16 knees from immature group 1 that were eligible for MRI evaluation with a T2-weighted oblique coronal slice, with the time-to-repetition (TR) of 2000 ms and time-to-echo (TE) of 103–110 ms. Eleven knees were excluded since they had undergone MRI under different TR and TE conditions. Similarly, a control group 2 with 32 knees (age, 25.6 ± 11.7; male, 16 and female, 16) was created by including data-matched controls from control group 1 who underwent MRI with the same sequence as those in immature group 2 (Fig. 1). Data matching was performed based on sex, body mass index (BMI) (± 2 kg/m^2^), and graft selection (semitendinosus tendon with or without gracilis tendon).

### ACL reconstruction and rehabilitation

Surgery was performed by a single senior orthopedic surgeon. An anatomical single-bundle ACLR with an HT graft was performed using a rounded rectangular femoral dilator, as previously reported^20^. Briefly, a femoral tunnel was created with a rounded rectangular shape at the center of the ACL’s anatomical femoral footprint by using rounded rectangular dilators with an inside-out technique. The long axis of the femoral tunnel was positioned with the intercondylar ridge as an anatomical landmark. The minor axis of the dilators was 6 mm, and the long axis had various sizes to match the graft size. The tibial tunnel was created using conventional drills, and a tibial guide was set at an angle of 50°; the tip of the aimer was positioned anteromedially and directed to the center of the tibial attachment of the ACL. The size of the tibial tunnel was decided based on the diameter of the graft using a conventional rounded sizing block. In all cases, the femoral and tibial tunnel locations were confirmed using fluoroscopy. Transphyseal ACLR was performed both in skeletally immature and mature patients. The HT graft was fixed using an adjustable length loop device at the femoral site and a double spike plate and screw (Smith and Nephew, Andover, USA) at the tibial site; the initial graft tension was set to 40 N at 20° of knee flexion.

Rehabilitation was also performed as previously reported^20^. Full range of motion and weight bearing were allowed depending on the patient’s pain on the day after surgery. Whenever meniscus repair was performed, the knee was immobilized with a brace for 2 weeks; partial weight bearing was allowed at 3 weeks, and full weight bearing was started at 4 weeks. Jogging and running were allowed at 3 months postoperatively if the quadriceps muscle strength was > 70% of that of the contralateral leg. A return to sports was allowed at 6 months after surgery if the quadriceps muscle strength was > 90% that of the contralateral leg, and if the physical assessment results for the half-squat test and single leg hop test were good. Biodex System 4 (Biodex Medical Systems, Shirley, NY, USA) was used to assess quadriceps muscle strength at 60°/s and 180°/s.

### Postoperative outcomes

We compared postoperative outcomes, including the incidence of revision, postoperative residual instability, and ACL injury on the opposite side between immature group 1 and control group 1 (Table 1). Postoperative residual instability was evaluated by the side-to-side difference in anteroposterior instability with KT-1000 (≥ 3 mm) or the pivot shift test (PST) at the final follow-up. These examinations were performed by a single orthopedic surgeon who was blinded to the MRI findings such as signal intensity of the graft. Patients were also assessed subjectively 1 year after surgery using the International Knee Documentation Committee (IKDC) knee score and Knee Injury and Osteoarthritis Outcome Score (KOOS)^21^, both of which have maximum and minimum scores of 100 and 0, respectively.

**Table 1:** Patient characteristics.

	Immature 1	Control 1	*P* value
Case (n)	27	175	
Age (years)	13.9 ± 1.2	26.2 ± 12.1	**0.001**
Sex (male: female)	11:16	85:90	0.488
Height (cm)	160.8 ± 7.5	165.4 ± 8.5	**0.001**
Weight (kg)	53.9 ± 8.5	63.6 ± 12.4	**0.001**
BMI (kg/m^2^)	20.7 ± 2.0	23.1 ± 3.3	**0.001**
Tegner activity scale (pre-operative state)	7.6 ± 1.5	6.9 ± 1.7	**0.049**
Tegner activity scale (postoperative state)	7.0 ± 1.7	6.4 ± 1.9	0.218
Graft (ST: STG)	13:14	87:88	0.880
Graft size on the femoral side	6 × 9 mm: 5	6 × 9 mm: 4	**0.001**
	6 × 10 mm: 20	6 × 10 mm: 139	
	6 × 11 mm: 2	6 × 11 mm: 30	
		6 × 12 mm: 2	
Lateral meniscus injury (n, %)	14/27 (63.0%)	83/175 (47.4%)	0.669
Medial meniscus injury (n, %)	10/27 (37.0%)	62/175 (35.4%)	0.871

A t-test, Mann–Whitney U, and the chi-square test were used.

*BMI* body mass index, *ST* semitendinosus tendon, *STG* semitendinosus tendon and gracilis tendon.

The bold values represent the significant difference, which p values were less than 0.05.

### Graft maturation

**Figure 2:**
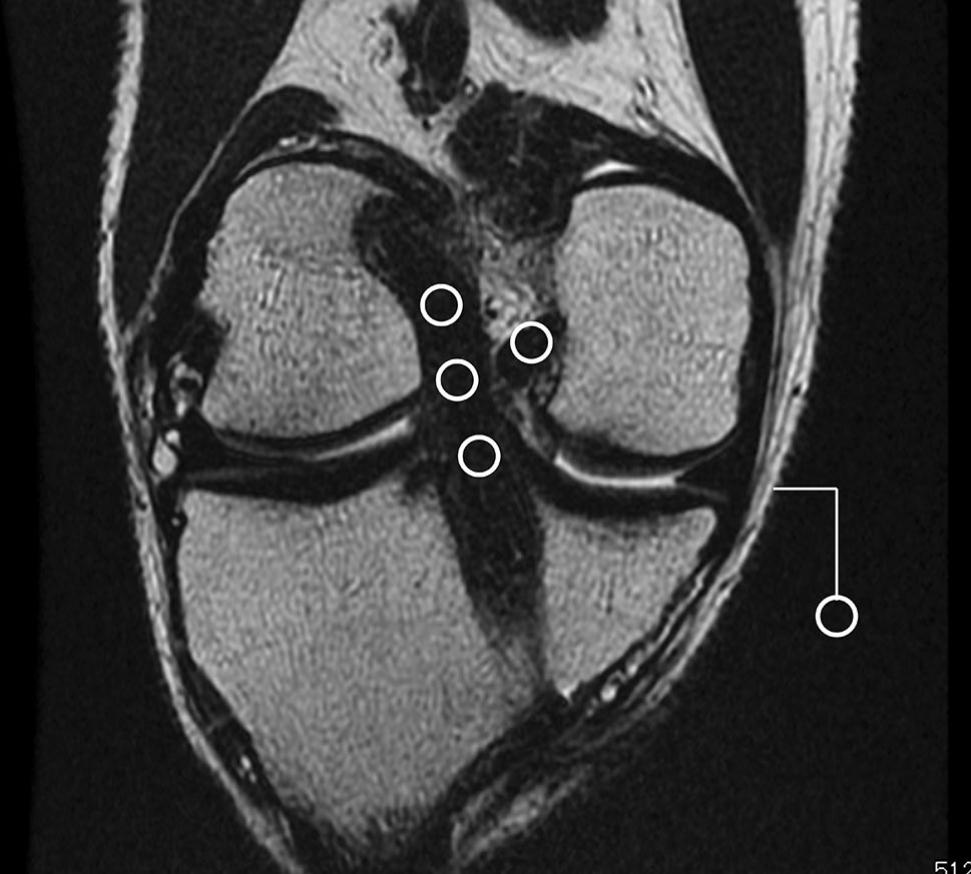
T2-weighted magnetic resonance image of the transplanted graft: oblique coronal slice. Graft signals at the 5 points (white circles) are calculated with circular regions of interest: three intra-articular sites of transplanted graft (proximal, mid-substance, and distal), posterior cruciate ligament, and background.

Graft maturation measurements were compared between immature group 2 and control group 2. Graft maturation was evaluated by measuring the graft signal of the reconstructed ACL on oblique coronal slices of T2-weighted conventional spin-echo MRI. Oblique coronal images with 2-mm thickness were created parallel to the reconstructed ACL, and the best single slice that demonstrated the full length of the ACL was used. A 1.5-T MRI scanner (Signa HDxt system, GE, Boston, USA) was used for image acquisition. Based on a previous study^22^, signal intensity was measured at five points with circular regions of interest (ROIs) (20 mm^2^): three intra-articular sites of the reconstructed ACL (proximal, mid-substance, and distal), posterior cruciate ligament, and background (Fig. 2). The background ROI was placed approximately 1 cm medial and 2 cm distal from the body surface at the level of the medial joint line. To normalize the signal intensities of the ACL grafts, the signal-to-noise quotient (SNQ) was calculated using the following equation^22,23^:

$$ {\text{SNQ}} = \left( {{\text{signal}}\;{\text{of}}\;{\text{reconstructed}}\;{\text{ACL}} - {\text{signal}}\;{\text{of}}\;{\text{the}}\;{\text{posterior}}\;{\text{cruciate}}\;{\text{ligament}}} \right){\text{/background}}\;{\text{signal}} $$

SNQ was evaluated by two orthopedic surgeons who were blinded to the surgery record and clinical outcomes.

### Statistical analysis

Data are presented as mean and standard deviation. The Shapiro–Wilk test was used to test the normality of data distribution. Age, body weight, BMI, pre- and postoperative state based on the Tegner activity scale, and subjective scores were compared using the Mann–Whitney U-test between the two groups. A t-test was used to compare the differences in the height and SNQ values between the two groups. The chi-square test was used to analyze categorical variables. To determine the risk of poor outcomes in skeletally immature patients, we calculated the odds ratio in the statistical analysis. Tukey’s honestly significant difference test was used to compare the SNQ values between the site of the reconstructed ACL within each group. A priori power analysis was conducted to determine the sample size to detect difference in SNQ values in a case–control study with an α-level of 0.05, a power of 0.8, and an allocation ratio of 2 using G-Power 3.1 software (Heinrich-Heine University Dusseldorf, Dusseldorf, Germany); the required sample size was 16 patients in immature group 2 and 32 patients in control group 2. The intra- and inter-observer reliability of measurements for the graft signal of proximal, mid-substance, and distal reconstructed ACL, determined using the intraclass correlation coefficient, was as follows: intra-observer reliability, ρ = 0.953, 0.862, and 0.897, respectively, which were almost perfect (ρ > 0.8)^24^; inter-observer reliability, ρ = 0.795, 0.863, and 0.793, respectively, which were almost perfect or substantial (ρ > 0.6)^24^. To assess intra-observer reliability, the graft signal on MRI was measured twice with an 8-week interval between measurements. A *P* value < 0.05 was considered significant. All data were analyzed using SPSS version 24.0 (SPSS; Chicago, IL, USA).

## Results

### Postoperative outcomes

The details regarding meniscal tear are represented in Table 2. All the patients with medial or lateral meniscal tears in immature group 1 underwent meniscal repairs. Regarding lateral meniscal tears in control group 2, 67 meniscal repairs and 9 meniscal resections were performed and 7 meniscal tears were left in situ without any procedure as they were stable. Regarding medial meniscal tears, 56 meniscal repairs and 5 meniscal resections were performed and one meniscal tear was left in situ without any procedure. A total of nine revision surgeries (9/202, 4.5%) and four contralateral ACLR (5/202, 2.5%) were performed in the study population. Indications for revision were re-tear during pre-injury sports (7/9, 77.8%), all of which were caused by non-contact injury and trauma (2/9, 22.2%). Arthroscopic findings revealed that all re-tear sites were proximal sites. All contralateral ACL injuries had occurred during a pre-injury sport. Among the nine re-tears following ACLR, six (66.7%) occurred within the first postoperative year. Conversely, three of the four contralateral ACL injuries occurred after the first postoperative year. Revision rates and postoperative PST positive rates were significantly higher in immature group 1 than in control group 1 (*P* = 0.020 and 0.003, respectively) (Table 3). There were no significant differences in the contralateral injury rates and anteroposterior instability between the two groups (*P* = 0.561 and 0.505, respectively). In contrast, subjective assessment of the IKDC score was significantly higher in immature group 1 than in control group 1 (94.3 ± 4.8 and 87.2 ± 13.1, respectively; *P* = 0.001). There was no significant difference in the KOOS of the two groups (96.0 ± 5.0 and 91.7 ± 9.0, respectively; *P* = 0.192).

**Table 2:** Details of meniscal tear.

	Lateral meniscus tear	Medial meniscus tear
**Immature group 1**		
Longitudinal	11	7
Radial	2	0
Bucket handle	1	2
Ramp lesion		5
**Control group 1**		
Longitudinal	46	38
Radial	30	0
Horizontal	0	1
Bucket handle	3	9
Flap	4	5
Ramp lesion		9

**Table 3:** Postoperative outcomes following anterior cruciate ligament reconstruction.

	Immature 1 (n = 27)	Control 1 (n = 175)	*P* value	Odds ratio	95% CI
Revision rate (n, %)	4/27 (14.8%)	5/175 (2.9%)	**0.020**	**5.913**	**1.480**–**23.622**
Opposite side injury (n, %)	1/27 (3.7%)	4/175 (2.3%)	0.516	n/a	
KT-1000 (≥ 3 mm) (n, %)	1/27 (3.7%)	11/175 (6.3%)	0.505	n/a	
Pivot shift test (n, %)	6/27 (22.2%)	7/175 (4.0%)	**0.003**	**6.857**	**2.105**–**22.339**

The chi-square test was used.

Odds ratio was analyzed in case of statistical analysis.

*CI* confidence interval.

The bold values represent the significant difference, which p values were less than 0.05.

### Graft signal

**Figure 3:**
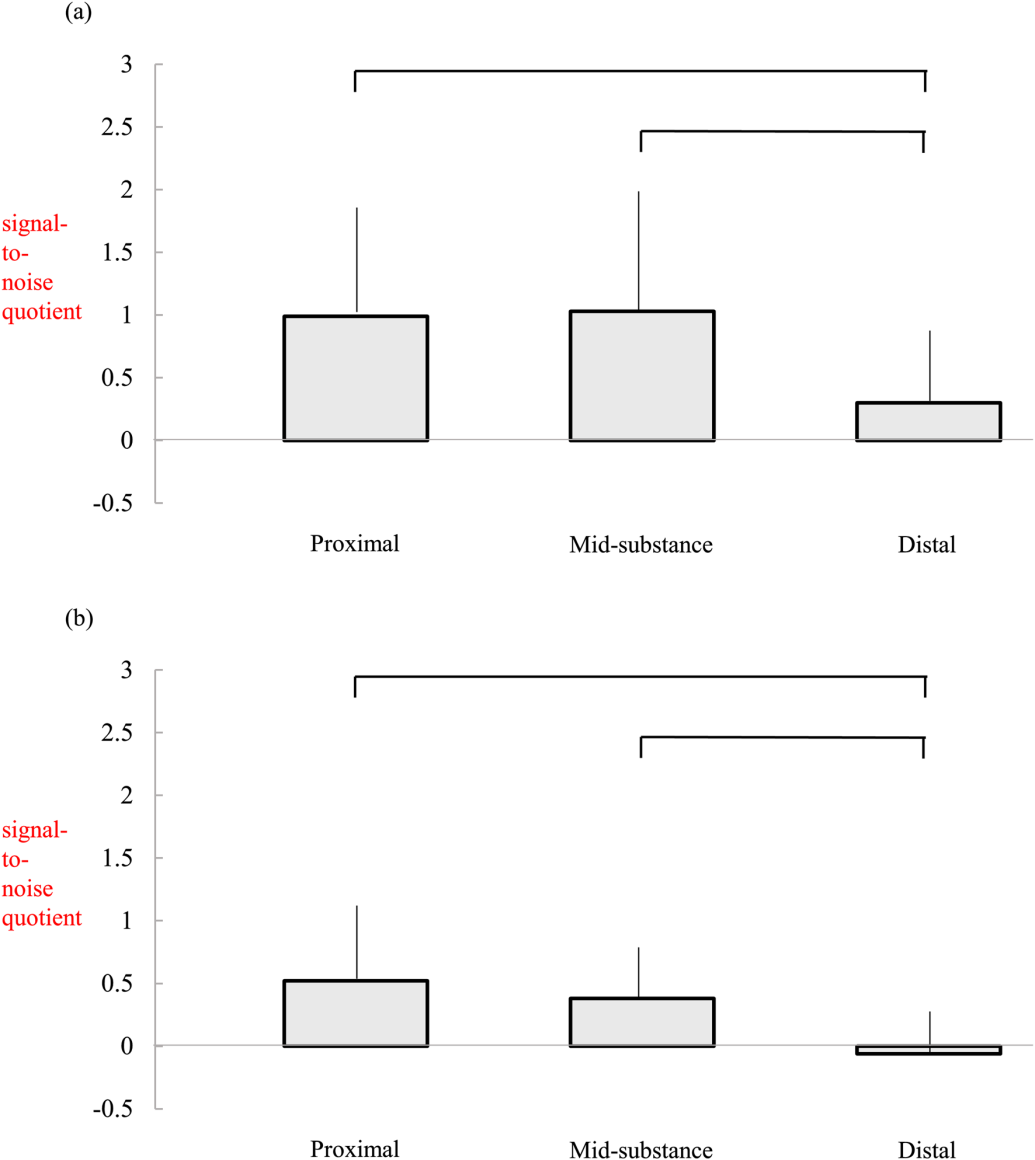
Comparison of the signal-to-noise quotients of the different reconstructed anterior cruciate ligament sites in the immature group 2 (**a**) and control group 2 (**b**). The SNQ of the distal site is significantly lower than that of the proximal and mid-substance sites within each group. *SNQ* signal-to-noise quotient.

SNQs in immature group 2 were significantly higher in the middle and distal regions of the reconstructed ACL than those in control group 2 at 6 months following ACLR (*P* = 0.003 and 0.034, respectively) (Table 4). Although SNQs in the proximal site of the reconstructed ACL in immature group 2 tended to be higher, no significant differences between the two groups were observed (*P* = 0.073). Within each group, SNQs of the distal sites were lower than those of the proximal or mid-substance sites (*P* values in immature group 2: proximal vs. mid-substance, 0.986; proximal vs. distal, 0.041; mid-substance vs. distal, 0.028) (*P* values in control group 2: proximal vs. mid-substance, 0.986; proximal vs. distal, 0.041; mid-substance vs. distal, 0.028) (Fig. 3).

**Table 4:** Comparison of signal-to-noise quotient of reconstructed anterior cruciate ligaments between the immature 2 and control 2 groups.

	Immature 2 (n = 16)	Control 2 (n = 32)	*P* value
SNQ on proximal site	0.99 ± 0.84	0.52 ± 0.58	0.073
SNQ on mid-substance site	1.03 ± 0.96	0.38 ± 0.41	**0.003**
SNQ on distal site	0.30 ± 0.56	− 0.06 ± 0.35	**0.034**

A t-test was used.

*SNQ* signal-to-noise quotient.

The bold values represent the significant difference, which p values were less than 0.05.

## Discussion

The principle finding of our study was that the SNQs of the reconstructed ACL in skeletally immature patients were higher than those in skeletally mature patients. This indicates that the graft in a skeletally immature patient develops less maturation over the 6-month period following ACLR with a rounded rectangular femoral tunnel than that in a skeletally mature patient. Moreover, the SNQ of the distal site was lower than that of other sites within each group. In clinical outcomes, a higher revision rate and residual rotational instability after ACLR with a rounded rectangular femoral tunnel were observed in the skeletally immature group.

In this study, a rounded rectangular femoral dilator was used to make a rounded rectangular femoral tunnel. An ACLR with a rounded rectangular femoral tunnel technique has the advantage of more favorable clinical outcomes and increased tendon-bone healing^17,18^. The clinical outcomes in this study supported those of previous study findings, which have confirmed poor outcomes following ACLR with the conventional round femoral tunnel in younger patients. A meta-analysis by Wong et al. revealed that there were 8.7% re-ruptures following ACLR among patients with a mean age of 13.0 years^25^. A cohort study from the Swedish and Norwegian knee ligament registries revealed that adolescents (13–19 years of age) have a 1.54–1.56 times increased risk of ACL revision compared with older patients^26^. Webster et al. reported a graft rupture rate of 18%, with half of the ruptures occurring in the first postoperative year in patients aged < 20 years^27^.

Early return to increased activity was a risk factor for postoperative re-tears. Geffroy et al. reported that 80% of young patients return to the same sport and about 60% return to the same sport at the same or higher level^4^. Moreover, higher rates of revision ACLR in young patients are considered to result from poor compliance with postoperative precautions^28,29^. As for subjective assessment, the IKDC in skeletally immature patients was higher than that in adults. However, there was no difference in KOOS between the groups. Magnitskaya et al. also showed that younger age had higher IKDC; however, age did not influence KOOS during the first year after surgery^21^. This finding indicates that younger patients may regain good knee motion and control of knee pain at 1 year postoperatively and possibly return to pre-injury level sports earlier than adults. We consider participation in high activity sports within the first postoperative year as a risk factor for graft re-tear and residual laxity. The proportion of skeletally immature ACLR patients who achieved > 90% of the limb symmetry index scores as that of the contralateral leg in four muscular strength tests (quadriceps, hamstrings, hip abductors, hip extensors) was reported to be only 20%, and 28% achieved good scores in some hop tests at 7 months following ACLR surgery^30^. Therefore, the timing of returning to sports for skeletally immature patients should be carefully considered^31^. Further, Ardern et al. claimed that pediatric rehabilitation must be used in close collaboration with the child’s parents/guardians^31^. Nevertheless, poor outcomes following ACLR in skeletally immature patients continue to be an unsolved problem.

The graft undergoes ligamentization following its implantation. Revascularization of the graft usually begins approximately 1 month following surgery, progresses from the periphery to the center of the graft by about 3 months, and finally, vascular density of the graft reaches that of the intact ACL during the remodeling phase^32^. Collagen fibers reorganize into fascicles during the remodeling phase; in this phase, the graft microscopically resembles the intact ACL^33^.

MRI is a useful non-invasive tool to assess graft ligamentization following ACLR. The SNQ on MRI, in particular, was the most common method reported in literature to assess graft ligamentization, wherein smaller values of the SNQ indicated better maturation of the reconstructed ACL^34^. The graft SNQ increases postoperatively, peaking at 4–6 months and then decreasing by 1 year^12^. Pauvert et al. reported that the graft SNQ was higher in the proximal site than in the middle and distal sites in patients with open growth plates^14^, which supports our study findings. They also observed a decrease in the graft SNQ from 6 months postoperatively to 12 months postoperatively, which reached a plateau phase at 24 months^14^. Several studies researched the correlation between SNQs and postoperative outcomes following ACLR^12,35^. Li et al. showed that a higher SNQ correlated positively with a high activity level, suggesting that more active patients are at a greater risk of graft failure^35^. Weiler et al. demonstrated a significant negative linear correlation between the SNQ and stiffness and tensile strength of the graft in a sheep model^13^. This supports our findings of a significantly higher SNQ in skeletally immature patients and their consequent clinical outcomes of higher revision and residual rotational instability rates following ACLR. Tissue degradation caused by vascular ingrowth that was detected as a higher SNQ may be an important factor contributing to the reduction in the graft tensile strength during the remodeling phase^36,37^. However, this finding contradicted that of previous studies that claimed the ACL-derived cells and environment in the immature skeleton as being more suitable for graft ligamentization process than that in the mature skeleton^38–40^. Meller et al. proposed that the immature tissue in younger patients may remodel faster and more completely than that in adults^38^. Magarian et al. researched human ACL tissue fibroblasts as a source of growth factors when stimulated by platelet concentrate. They observed that immature cells in younger subjects showed a higher ability to migrate and proliferate than mature cells^39^. Nakano et al. observed that ACL-derived cells from younger subjects enhanced early bone-tendon healing in an immunodeficient rat model of ACL reconstruction^40^. These findings suggest younger age as an advantage for graft ligamentization. Therefore, there may be some other factors influencing higher SNQ and poor outcomes in skeletally immature patients.

The microenvironment surrounding the graft plays an important role in the ligamentization process^9,41^. Furthermore, skeletally immature patients have been shown to have significantly higher levels of Resolvin E1 and IL-10 and showed different inflammatory pattern compared with adults with ACL lesions^42^. In this study, the SNQ of the distal site was lower than that of the proximal or mid-substance sites. This result is supported by that of a previous study, which reported that the mean SNQ of the femoral intraosseous graft was significantly higher than that of the tibial side or the tibial intraosseous graft^10^. This could be secondary to repetitive stress due to abrasive forces on the graft at the femoral tunnel aperture, wherein the graft is acutely bent and stretched, causing graft damage and preventing graft healing in the early postoperative phase^43,44^. Tohyama et al. observed an association between an excessive increase in tendon stress and a reduction in tensile strength in the early postoperative phase^45^. Hence, the initial mechanical strength of the graft prior to transplantation appears to be a crucial factor. Several studies have shown that the fibril diameter increases from young age to adulthood, decreases from adulthood to elderly, and correlates with stiffness and strength in animal models^46,47^. The tendon in skeletally immature patients may be mechanically weaker than that in mature patients and may be unable to withstand repetitive bending and stretching stress. Therefore, age-related histological changes in the transplanted tendon may influence higher SNQ values and poorer outcomes following ACLR. Further studies regarding the optimal graft choice for treatment of skeletally immature patients are warranted. Dabis et al. have reported that ACL repair may be a good choice for proximal ACL tears in pediatric patients^48^. Moreover, re-ACL injury in skeletally immature patients requires a careful graft choice, such as iliotibial band^49^.

This study has some limitations. First, we could not avoid selection bias in the choice of subjects in control group 2. Although the sample size was based on power calculations, there were only 16 patients in immature group 2 who were evaluated using MRI under the required conditions. Second, the SNQ was evaluated only at 6 months following surgery and not at multiple time points to assess the rate of graft remodeling. We do not know exactly when a skeletally immature patient’s graft begins to take the appearance of a skeletally mature patient’s graft. However, we were more interested in differences in the SNQ between the two groups at the time when patients consider returning to sports. Third, our follow-up duration was relatively short. This study did not evaluate postoperative complications such as leg-discrepancy or misalignment in skeletally immature patients. However, since several graft failures occur within the first year following surgery^26,27^, our follow-up period might have been adequate to assess the revision rates. Fourth, although all patients received similar surgical procedures and rehabilitation, there were inadequate data regarding the factors that may have influenced postoperative recovery, such as restricting participation in sports or exercise at home. Poor compliance with postoperative rehabilitation in younger patients may have affected their postoperative outcomes. Fifth, although the graft size was different between two groups, the influence of graft size was not assessed in this study; however, the graft size may influence the outcomes following ACLR.

Ligamentization of the reconstructed ACL is a very important factor that influences the outcomes following ACLR. There are several factors to explain why the skeletally immature group had a higher failure rate. In this study, it was confirmed that there was a difference in the SNQ of the reconstructed ACL between skeletally immature and mature patients. Therefore, maturation of the reconstructed ACL may be a key factor for poor outcomes following ACLR in skeletally immature patients.

## Conclusions

Skeletally immature patients were observed to have higher graft revision and residual rotational laxity rates following ACLR with a rounded rectangular femoral tunnel. The reconstructed ACL in skeletally immature patients showed higher signal intensity on MRI at 6 months postoperatively.
